# The complete chloroplast genome of *Indosasa hispida* ‘Rainbow’ (Poaceae, Bambuseae): an ornamental bamboo species in horticulture

**DOI:** 10.1080/23802359.2021.1994478

**Published:** 2022-04-04

**Authors:** Dandan Tu, Chaomao Hui, Liyue Zhu, Wenjun Zhang, Weiyi Liu

**Affiliations:** a Sympodial Bamboos Technological and Engineering Research Center (SymBTERC) National Forestry and Grassland Administration (NFGA), Southwest Forestry University, Kunming, China; b Key Laboratory for Forest Resources Conservation and Utilization in the Southwest Mountains of China, Ministry of Education, Southwest Forestry University, Kunming, China

**Keywords:** *Indosasa hispida* ‘Rainbow’, chloroplast genome, phylogenomic

## Abstract

*Indosasa hispida* ‘Rainbow’ is a new horticultural plant variety for anthocyanin production, which has great ornamental value and huge market potential. The chloroplast genome is 139,690 bp in length, containing a large single-copy region (LSC) of 83,268 bp, a small single-copy region (SSC) of 12,830 bp, and a pair of 21,796 bp inverted repeats region (IR). The GC content of chloroplast genome is 38.9%. There are 130 genes in the cp genome, including 83 protein-coding genes, 8 ribosomal RNA genes, and 39 transfer RNA genes. In addition, phylogenetic analysis firmly supported that *I. hispida* 'Rainbow' constituted that a sister species with *Pleioblastus maculatus*.

*Indosasa hispida* McClure ‘Rainbow’ Y. M. Wang et J. Wang 2012 is a plant of the genus *Indosasa* McClure of the grass family bamboo subfamily. It is a scattered shrub-like small bamboo, which is a new horticultural plant variety introduced and bred from the mutant plants of *I. hispida* McClure. The characteristics of *I. hispida* ‘Rainbow’ is similar to those of the mother bamboo *I. hispida* McClure, the key difference lies in that the culms of *I. hispida* ‘Rainbow’ is 2–3 m high and 1.0–3.0 cm in diameter. The culms are covered with small bristles on the surface when they are young, and then fall off and become hairless, with white powder below the nodes; its culm presents distinctive red to fuchsia because of the abundance of Anthocyanins, and its leaves with white to light yellow stripes. *I. hispida* ‘Rainbow’ is originated **in the** tropical and southern subtropical mountains below 1000 m above sea level in Jinghong, Mengla and Pu’er, Jiangcheng, Xishuangbanna, southern Yunnan. *I. hispid*a ‘Rainbow’ as a rare colored bamboo genus, it has highly innovative significance of the application of landscape ornamental (Wang et al. 2015). Although the growth habits and the cultivation techniques of *I. hispida* ‘Rainbow’ had been reported (Ma et al. 2015), complete chloroplast genome of this bamboo has not been reported. In this study, we obtained the chloroplast complete genome, which will provide useful data onto phylogeny research and genomic selective breeding of *I. hispida* ‘Rainbow’.

Total genomic DNA was extracted from fresh leaves of *I. hispida* ‘Rainbow’ with Rapid Plant Genomic DNA Isolation Kit (BALB, Beijing, China), which were collected from Southwest Forestry University (25°6′30′′N, 102°45′23′′E), China. The DNA and specimen (accession number: SWFU1992020) were deposited at the Sympodial Bamboos Technological and Engineering Research Center (SymBTERC) National Forestry and Grassland Administration (NFGA), Southwest Forestry University, Kunming, China (Website:http://symb.swfu.edu.cn/, Contact: Liyue Zhu, Email: 2465815340@qq.com), and the paired-end library was prepared and sequence in Sangon Biotech, Shanghai, China, which gained 3.9 Gb of average150 bp paired-end raw reads. The chloroplast genome was assembled by using the program NOVOPlasty (Dierckxsens et al. 2017). And the program PGA (Qu et al. 2019) was applied to annotate the chloroplast genome with *I. silica* (GenBank accession MH394382) cp genome as the reference.

The complete *I. hispida* ‘Rainbow’ (GenBank accession: MW463058) chloroplast genome is composed of a large single-copy region (LSC) of 83,268 bp, a small single-copy region (SSC) of 12,830 bp, and two inverted repeats (IR) region of 21,796 bp, which is a circular molecule with a total length of 139,690 bp. The total GC content of the whole genome is 38.9%, and corresponding values of LSC, SSC, and IR regions were 36.97%, 33.34%, and 44.23%, respectively. The cp genome contains 130 genes, including 83 protein-coding genes, 8 rRNA genes, and 39 tRNA genes. Fifteen genes contain introns, of which *trnK, rps16, trnG, atpF, trnL, trnV, petB, petD, rpl16, rpl2, ndhB, trnI, trnA, ndhA* each contain one intron, *ycf3* consists of two introns.

Phylogenetic analysis was carried out based on the 29 complete chloroplast genomes in subfamily Bambusoideae downloaded from the NCBI GenBank database, for purposes of finding the phylogenetic location of *I. hispida* ‘Rainbow’. The sequences were aligned by MAFFT v7.450 (Rozewicki et al. 2019). And we constructed the phylogenetic tree (maximum likelihood) based on K3Pu + F + R5 model in RAxML-NG v0.90 (Kozlov et al. 2019), and calculated bootstrap probability values from 1,000 replicate. In general, the sequence alignment phylogenetic analyses demonstrated that *I. hispida* ‘Rainbow’ was highly clustered in temperate woody bamboo with *I.sinica* and *I. shibataeoides* in the same genus(Ma et al. 2017). However, We compared the sequence homologies of I. hispida ‘Rainbow’ and its sister species *Pleioblastus maculatus* by Blast software, and found that the similarity was 99.92%, and the similarity between *I. hispida* ‘Rainbow’ and other Indosasa species ranged from 99.88% to 99.91%. *I. hispida* ‘Rainbow’ is not clustered on the same branch as *I. sinica* and *I. shibataeoides* to form a monophyletic group, being sister to the clade of *Pleioblastus maculatus*, and has a high approval rating (ML = 92%) (Figure 1). This situation may be the result of convergent evolution and parallel evolution, although there are similarities in characters between *I. hispida* ‘Rainbow’ and the other two *Indosasa* species, the character states of different species may not be inherited from the common ancestor, is produced by multiple autonomous evolutions.

## Data Availability

My data have been uploaded to GenBank (https://www.ncbi.nlm.nih.gov/nuccore/MW463058), GenBank Accession: MW463058. And Rawdata has been uploaded to SRA(https://submit.ncbi.nlm.nih.gov/subs/sra/SUB9018892/overview), BioProject accessions: PRJNA698728, BioSample accession: SAMN17734893, SRA accession: SRR13651372. The maximum likelihood tree is constructed from 30 chloroplast genomes of Bambuseae. Numbers near the node are displayed as bootstrap support values.
